# Adaptation and Prosthesis Effects on Stride-to-Stride Fluctuations in Amputee Gait

**DOI:** 10.1371/journal.pone.0100125

**Published:** 2014-06-23

**Authors:** Shane R. Wurdeman, Sara A. Myers, Adam L. Jacobsen, Nicholas Stergiou

**Affiliations:** 1 Biomechanics Research Building, University of Nebraska at Omaha, Omaha, Nebraska, United States of America; 2 Advanced Prosthetics Center, Omaha, Nebraska, United States of America; 3 Veterans Affairs Medical Center, Omaha, Nebraska, United States of America; University of California, Merced, United States of America

## Abstract

Twenty-four individuals with transtibial amputation were recruited to a randomized, crossover design study to examine stride-to-stride fluctuations of lower limb joint flexion/extension time series using the largest Lyapunov exponent (λ). Each individual wore a “more appropriate” and a “less appropriate” prosthesis design based on the subject's previous functional classification for a three week adaptation period. Results showed decreased λ for the sound ankle compared to the prosthetic ankle (F_1,23_ = 13.897, *p* = 0.001) and a decreased λ for the “more appropriate” prosthesis (F_1,23_ = 4.849, *p* = 0.038). There was also a significant effect for the time point in the adaptation period (F_2,46_ = 3.164, *p* = 0.050). Through the adaptation period, a freezing and subsequent freeing of dynamic degrees of freedom was seen as the λ at the ankle decreased at the midpoint of the adaptation period compared to the initial prosthesis fitting (*p* = 0.032), but then increased at the end compared to the midpoint (*p* = 0.042). No differences were seen between the initial fitting and the end of the adaptation for λ (*p* = 0.577). It is concluded that the λ may be a feasible clinical tool for measuring prosthesis functionality and adaptation to a new prosthesis is a process through which the motor control develops mastery of redundant degrees of freedom present in the system.

## Introduction

Lower limb amputation presents a major change to the patient's neuromuscular system. The loss of peripheral structures and neural endpoints creates an obstacle for the individual as they potentially learn to walk again following prosthetic rehabilitation. The neuromuscular system must learn new strategies in order to fully integrate a foreign device into its natural movement pattern. Consider prior to amputation, during the common task of walking, the neuromuscular system had developed a movement strategy that encompassed an active, biological leg. Following amputation, major components of the anatomy that led to the solution that the neuromuscular system had settled on are no longer present, thereby leaving the neuromuscular system to learn a new solution if the person is to walk again with a prosthesis. The need for the neuromuscular system to learn a new solution is not unique to limb loss, but occurs under many different pathologies affecting the neuromusculoskeletal system [1].

Contrary to other pathologies that affect the neuromuscular system's previous solution to the multiple variables involved in the task of walking, individuals with a prosthesis will find their motor control being challenged to re-learn every time a new prosthesis is introduced. A new prosthesis will change the variables that the neuromuscular system is accounting for in order to resolve upon the appropriate solution. Importantly, the movement solution that results will manifest within the subtle stride-to-stride fluctuations that are naturally occurring over multiple strides [1]. Perhaps not surprising then, previous work has indeed found altered stride-to-stride fluctuations when walking for individuals with a unilateral, transtibial prosthesis compared to their healthy counterparts [2]. More specifically, Wurdeman et al.[2] reported an increased largest Lyapunov exponent (λ) for motion about the prosthetic ankle as well as the sound leg hip and knee. The λ is a measure of stride-to-stride fluctuations that examines the rate dependent divergence of nearby points within an attractor, representing how quickly a point will vary from stride-to-stride [2]–[6].

Consistent with any learning task where certain things are naturally easier to learn than others, some prostheses will present variables that will make it easier for the neuromuscular system to determine a solution. On the other hand, other prostheses may present too many variables or the variables presented by the new prosthesis may be too different from those that were naturally occurring or accounted for in a previous prosthesis. Either of these scenarios could lead to a poor solution by the neuromuscular system as it attempts to accomplish the task of walking. A poor solution may be the reason that when presented with a new prosthesis, which altered the stride-to-stride fluctuations during walking, individuals with an amputation exhibited a prosthesis preference that was strongly correlated to the λ such that they preferred the device that resulted in a reduced λ [4].

Yet, what is unknown, is the behavior that will result when individuals with amputation are asked to learn to use a new device. Adaptation to a prosthesis is the period of time through which learning and ultimately a movement solution is discovered. The gravity of such a learning period is such that it is often cited as a limitation in many prosthetics studies [7]–[14]. A better understanding of how individuals modify their behavior (i.e. changes in stride-to-stride fluctuations) throughout the adaptation period could provide understanding of what could be expected in terms of outcomes. Even more importantly, an understanding of adaptation provides insight into the process by which the neuromuscular system is able to resolve all the potential movement strategies into a single, optimal solution [15], [16]. Such insight could potentially help guide future rehabilitation strategies to optimize outcomes.

Therefore, the purpose of this study was to examine the effects of an adaptation period on stride-to-stride fluctuations in both the sound leg and prosthetic leg following receipt of a new prosthesis. It was hypothesized as the individual's neuromuscular system learns to fully integrate the prosthesis into the person's movement, there will be a decrease in stride-to-stride fluctuations as the movement converges on a solution more similar to healthy, non-amputees [2]. Furthermore, if a new prosthesis presents variables that allow the individual's neuromuscular system to settle into its natural movement solution this will intuitively result in decreased stride-to-stride fluctuations (i.e. more similar to their healthy counterparts [2]). On the other hand, if the new device presents variables that are very foreign to those that the neuromuscular system would naturally incorporate into its innate movement strategy, then increased stride-to-stride fluctuations would be expected (i.e. less similar to their healthy counterparts [2]). Therefore, it was also hypothesized that a more appropriate prosthesis design would result in decreased stride-to-stride fluctuations compared to a less appropriate prosthesis design.

## Methods

### Participants

Twenty-four individuals (19 males, 5 females) with unilateral, transtibial amputation were recruited for this study (Table 1). The study was approved by the University of Nebraska Medical Center IRB "Nonlinear Analysis of Amputee Gait", 021-11-EP, and by the Nebraska/Western Iowa Veterans Affairs Medical Center IRB "Nonlinear Analysis of Amputee Gait", 00793. All participants provided written informed consent as approved by the overseeing Institutional Review Boards. Inclusion criteria included: 1) ability to ambulate non-stop for three minutes, 2) able to commit to a 6 week protocol, and 3) have had their current prosthesis longer than thirty days. Exclusion criteria included: 1) presence of any ulcers on either the residual limb or contralateral limb, 2) inability to provide informed consent due to cognitive condition, 3) exoskeletal type prosthesis or non-removable cosmetic cover (prevents exchanging of components without destroying person's prosthesis), 4) presence of any major neuromuscular or musculoskeletal conditions affecting gait (i.e. stroke, Parkinson's disease, multiple sclerosis), 5) previously classified by physician as K1 or K0 level ambulatory [17], or 6) a poor fitting current prosthesis.

**Table 1:** Subject demographics. Note all participants were MFCL K3 or K4 level ambulators.

Age (yrs)	Height (cm)	Mass (kg)	Time Since Amputation (yrs)	Self-selected speed (m/s)	Residual limb length (cm)	Cause of amputation
53.3 (11.6)	177.6 (7.9)	100.8 (18.4)	8.7 (9.9)	0.85 (0.39)	15.7 (3.6)	14 trauma, 7 vascular/diabetes, 1 cancer, 2 infection

Mean (SD).

### Procedures

Subjects participated in a 6 week, randomized-crossover design adaptation protocol. This encompassed two separate 3 week adaptation periods [18]. All prosthesis modifications and data collections occurred within the University's gait laboratory. At the initial visit, the subject's foot/ankle/pylon were removed distal to the socket in preparation for a different prosthesis. For the duration of the study, subjects wore the socket that their own prosthetist had created for them as well as utilizing their own current method of suspension. Once the foot/ankle/pylon were removed, an alternate foot/ankle/pylon were assembled and attached. The alternate prosthesis design was classified as either “more appropriate” or “less appropriate” based on the prosthesis activity level and the subject's previously determined activity/functional level. In other words, if a subject was classified as a K3 ambulator, then the prosthesis setup utilizing the K3 level foot (high activity) would be considered “more appropriate”, whereas the prosthesis setup with the K2 level foot (low activity) would be deemed “less appropriate”. The prosthesis was then aligned by a certified prosthetist. Once the prosthesis was properly aligned, the initial gait analysis was performed. Subjects then wore the device home and returned in 1.5 weeks to complete another data collection. After 3 weeks of wearing the alternate prosthesis, subjects again returned for a final data collection with the initial alternate prosthesis. Following the data collection with the initial alternate prosthesis, the foot/ankle/pylon sections were again removed and again an alternate foot/ankle/pylon were assembled and attached. The second alternate prosthesis setup was different from the initial setup; if the first prosthesis was “more appropriate”, then the second prosthesis was the “less appropriate” or vice versa. Order for prosthesis type was randomized across subjects. The prosthesis was again re-aligned based on its current setup by a certified prosthetist. The wear and data collection procedures were then repeated similar to the initial prosthesis. This resulted in three data collections per prosthesis per subject.

The same procedure was utilized for all data collections. Subjects performed 2 separate walking trials on a treadmill. Each trial was 3 minutes non-stop at their self-selected preferred walking speed with at least 1 minute rest between trials to avoid fatigue. The walking speed was determined at the initial visit with the same speed subsequently utilized for all walking trials. Subjects were permitted to use the hand rail if needed for balance but were instructed not to place weight through their arm. Subjects wore a tight fitting uniform during all walking trials. Twenty-seven retro-reflective markers were placed on various anatomical locations on the lower limbs [2], [4], [19] such that each segment had a minimum of three non-collinear markers to allow three dimensional relative joint angle calculations. On the prosthetic limb, markers were placed on analogous locations as the sound limb. Marker motion was recorded in three dimensions with a 12 camera motion capture system at 60 Hertz (Motion Analysis Corp., Santa Rosa, CA, USA). Lower limb joint angle flexion/extension time series for each joint of the sound and prosthetic limbs were then calculated from the raw marker position data (Visual 3D, Germantown, MD, USA).

### Analysis

Stride-to-stride fluctuations were calculated using λ. The λ is a measure of how quickly similar points in state space diverge along their respective trajectories [2]–[4]. In terms of gait, it represents how quickly an independent point in the gait cycle fluctuates from other similar points in the gait cycle occurring during a different stride. If the walking pattern were perfectly periodic, then two points occurring at the same point in the gait cycle would then have similar successive points. In gaits that have more stride-to-stride fluctuations, the two points occurring at the same point in the gait cycle would then have very different successive points due to large fluctuations. The λ is chosen specifically for its ability to detect stride-to-stride fluctuations that are overlying a strongly periodic movement. Joints flex and extend repeatedly with every stride during controlled walking. This repeated motion is not perfectly similar with every cycle, but the motion is similar enough such that other measures (e.g. detrended fluctuation analysis, sample entropy, approximate entropy, etc.) potentially examining stride-to-stride fluctuations in the joint motions may algorithmically have their end calculation dominated by this strong, underlying periodicity. The method for the calculation of the λ is outlined in greater detail in previous studies [2], [4]. For the adaptation portion of the study, all joint angle flexion/extension time series were subsequently cropped to 110 strides with the lone exception of 1 subject that was only able to attain 70 strides in all data collections. Trials were cropped to 110 strides as this was the maximum amount that the individual who took the least strides was able to achieve with the lone exception of the individual that took 70. This individual's walking trials were therefore cropped to 70 strides. The large discrepancy between this individual and the other 23 subjects was the reason for not cropping all trials to 70 strides. Furthermore, the study utilized a within subject design and thus a similar number of strides are being compared for each subject. The embedding dimension and time lag for each time series were calculated using the false nearest neighbor and average mutual information algorithms, respectively [2], [20], [21]. All time series were consequently embedded with the average dimension of 7. The λ was then calculated for each joint of the sound and prosthetic legs. Of note, only the first treadmill trial was used for analysis unless during the data collection or in post-processing problems were noted (e.g. subject's foot clipped the side of the treadmill or large marker dropouts during trial resulting in excessive interpolating of marker position data). In these cases the second trial was utilized for analysis. Calculation of λ requires several input parameters which were set to the following: time evolution equal to 3 [2], [4], [22], max angle to replacement point equal to 0.3 radians [2], [4], [22], minimum scale length of 0.0001 [2], [4], [22], and maximum scale length of 0.1 times the maximum diameter of the attractor (maximum distance to selection of new nearest neighbor) [2], [4], [22]. Main effects for leg (prosthetic vs. sound), prosthesis (more vs. less appropriate), and adaptation (visit 1 vs. visit 2 vs. visit 3) at the hip, knee, and ankle were tested through a 2×2×3 fully repeated ANOVA (α = 0.05) with Fisher's LSD for post-hoc. An analysis of trend was performed for adaptation effects through the course of the 3 weeks. All statistical analyses were done using SPSS (SPSS 16.0. Chicago, IL, USA).

## Results

**Figure 1:**
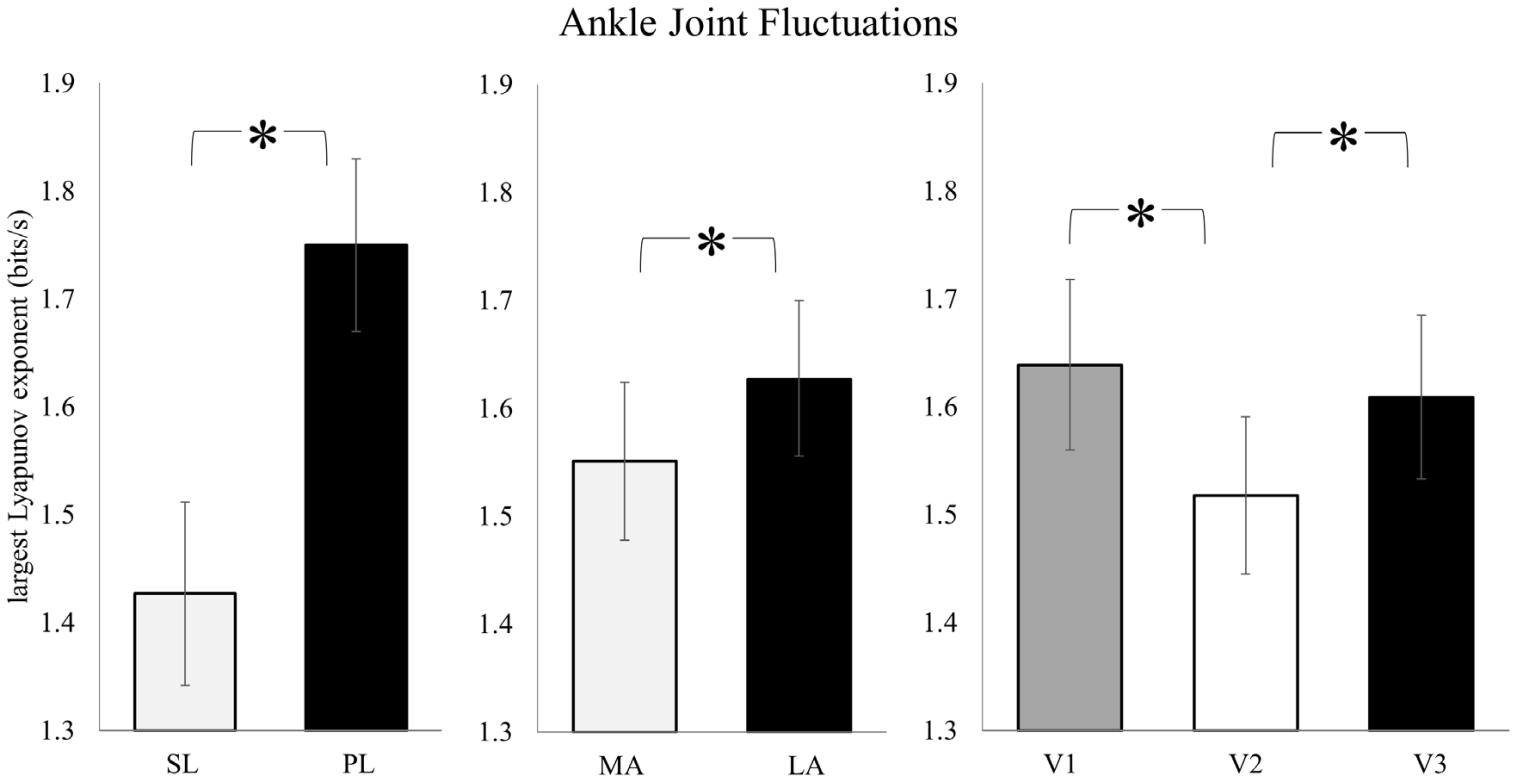
Stride-to-stride fluctuations for the ankle were significantly decreased for the sound leg compared to the prosthetic leg. The “more appropriate” prosthesis design also yielded decreased stride-to-stride fluctuations compared to the “less appropriate” prosthesis. Through the adaptation, a significant U-shaped quadratic trend was present, with significantly increased stride-to-stride fluctuations at the initial visit and final visit compared to the middle of the adaptation period. (mean ± SEM) SL: sound leg; PL: prosthetic leg; MA: “more appropriate” prosthesis; LA: “less appropriate” prosthesis; V1: initial visit; V2: second visit; V3: final visit. *Sig. at *p*<0.05.

At the ankle, there were significant main effects for leg, prosthesis, and visit (Figure 1). The sound leg ankle had significantly reduced λ compared to the prosthetic ankle (F_1,23_ = 13.897, *p* = 0.001) with an observed power of 0.946. The “more appropriate” prosthesis resulted in reduced λ when compared to the “less appropriate” prosthesis design (F_1,23_ = 4.849, *p* = 0.038) with an observed power of 0.559. For visit there was also a significant effect (F_2,46_ = 3.164, *p* = 0.050) with an observed power of 0.578. Post-hoc analysis showed the initial visit (i.e. initial fitting) to have a significantly increased λ compared to the second visit (i.e. middle of 3 week period; *p* = 0.032), and the final visit (i.e. end of adaptation period) had a significantly increased λ compared to the second visit (*p* = 0.042). The λ values for the initial and final visits were not statistically different (*p* = 0.577). This yielded a significant U-shaped quadratic trend across the adaptation period (*p* = 0.013). There were no significant interactions.

**Figure 2:**
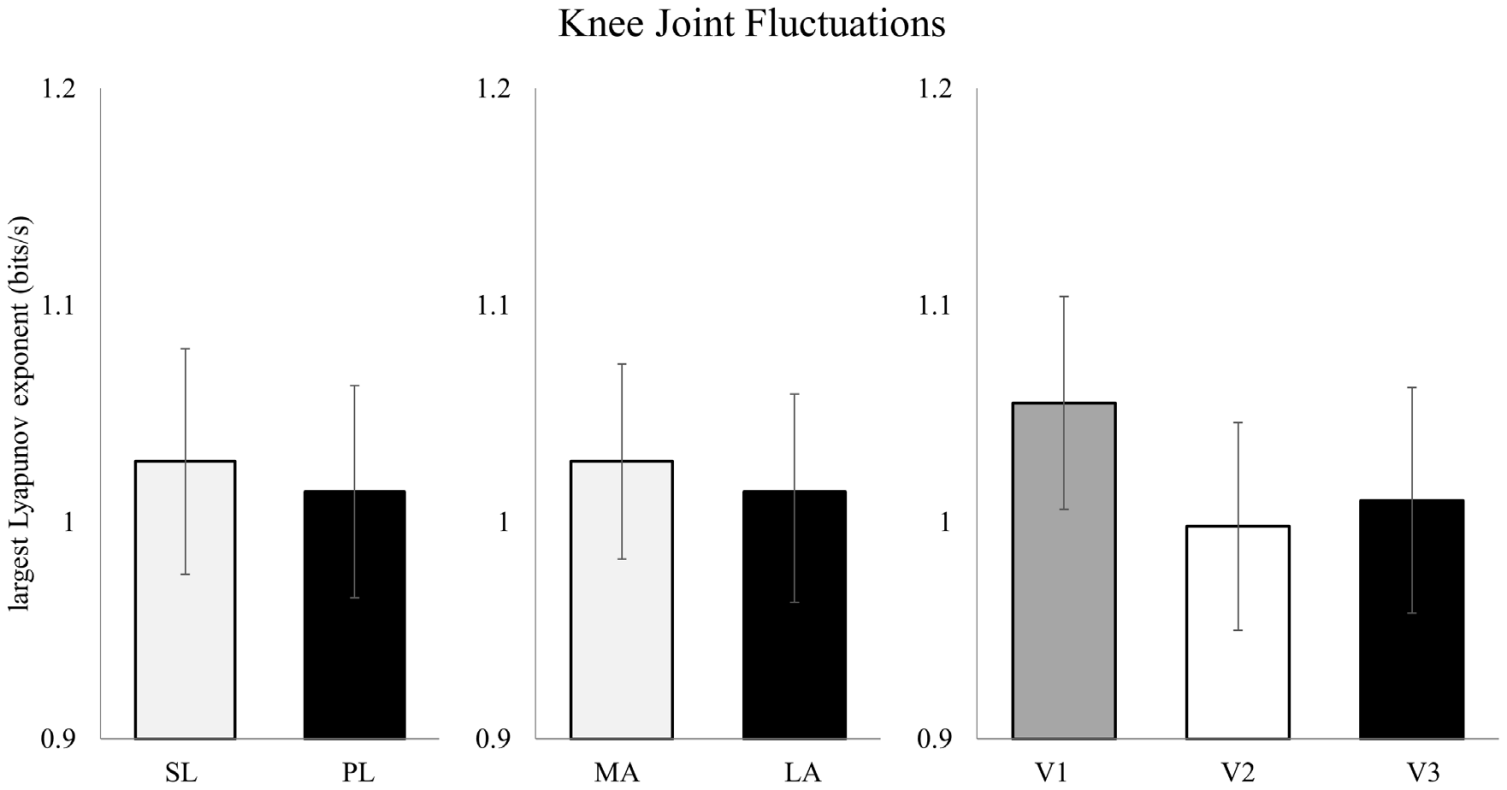
Differences in stride-to-stride fluctuations for the knee were not significant for the effect of leg, prosthesis, or time point in the adaptation period. (mean ± SEM) SL: sound leg; PL: prosthetic leg; MA: “more appropriate” prosthesis; LA: “less appropriate” prosthesis; V1: initial visit; V2: second visit; V3: final visit. *Sig. at *p*<0.05.

At the knee, there was no significant effect for leg (sound vs. prosthetic; F_1,23_ = 0.149, *p* = 0.703; Figure 2). There was also no significant effect for prosthesis (“more appropriate” vs “less appropriate”; F_1,23_ = 0.387, *p* = 0.540), or for visit (F_2,46_ = 2.402, *p* = 0.102). There were no significant interactions.

**Figure 3:**
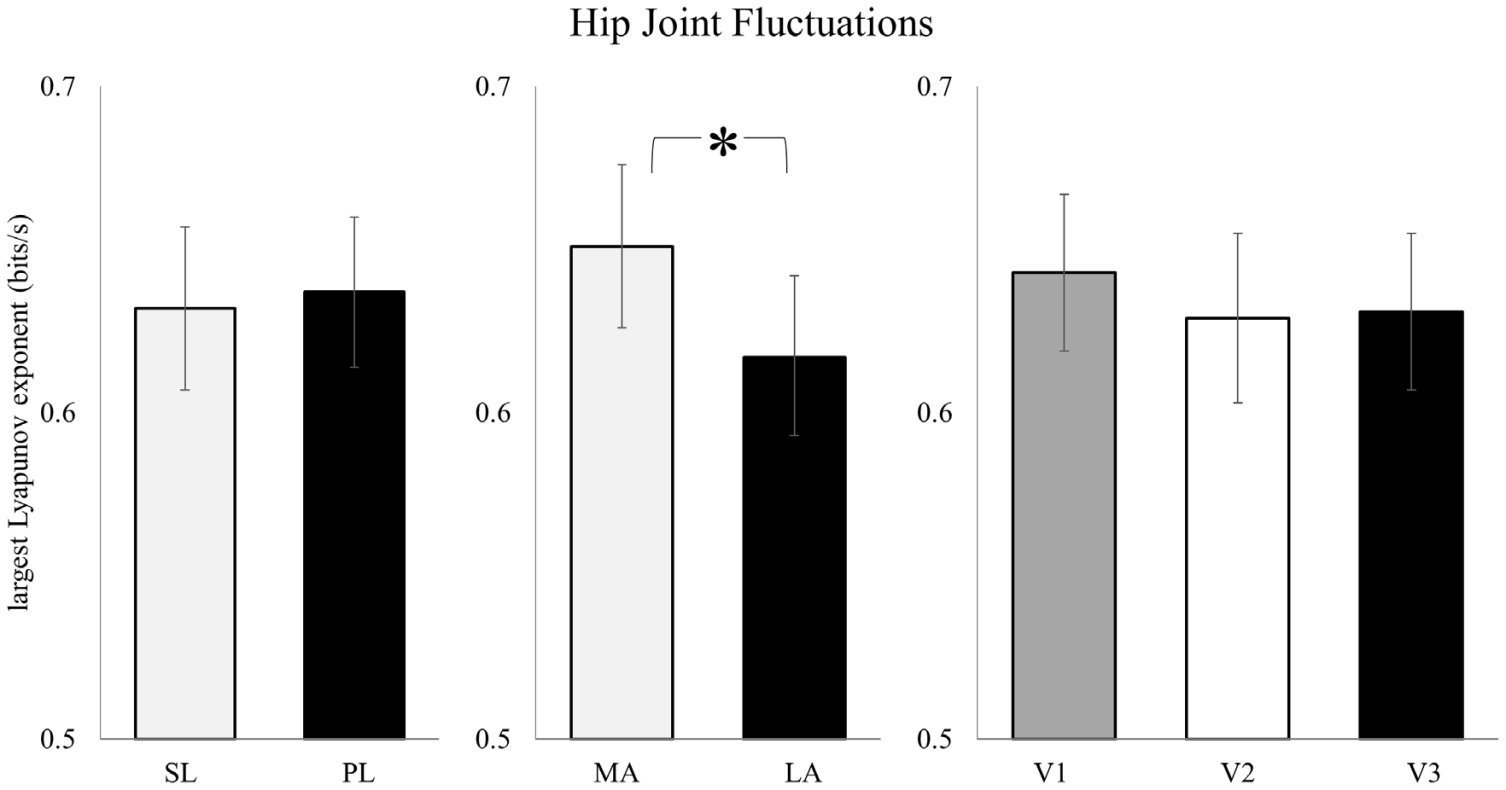
Stride-to-stride fluctuations for the hip were not significantly different for the effect of leg or for changes across the adaptation period. The “more appropriate” prosthesis design did however result in increased fluctuations at the knee compared to the “less appropriate” design. (mean ± SEM) SL: sound leg; PL: prosthetic leg; MA: “more appropriate” prosthesis; LA: “less appropriate” prosthesis; V1: initial visit; V2: second visit; V3: final visit. *Sig. at *p*<0.05.

Similar to the knee, the hip showed no effect for leg (F_1,23_ = 0.187, *p* = 0.669) or for visit (F_2,46_ = 0.681, *p* = 0.511). This was not the case, however, for prosthesis. Counter to the ankle, the “more appropriate” prosthesis design had an increased λ compared to the “less appropriate” design (F_1,23_ = 5.300, *p* = 0.031; Figure 3), with an observed power of 0.597. There were no significant interactions.

## Discussion

The primary significant findings occurred at the ankle. This is not entirely surprising given previous work showing a significantly increased λ at the prosthetic ankle compared to the sound ankle and compared to healthy control ankles [2]. In addition, it was only the λ at the prosthetic ankle that was previously found to be strongly correlated with the patient's prosthesis preference [4]. Our results with a larger sample size comparing the λ between the prosthetic ankle and the sound leg ankle agree with previous work by Wurdeman et al. [2]. More specifically, the motion about the prosthetic ankle has increased stride-to-stride fluctuations compared to the sound ankle. This would seem to continue to highlight the motion of the prosthetic ankle as a primary signal of the effectiveness of the person's motor control. It has previously been stated that the λ for the motion about the prosthetic ankle represents the union of the biological system (i.e. amputee) and the mechanical system (i.e. prosthesis) in an effort to work cooperatively as a single amputee-prosthesis locomoting system [4]. This is believed to be the case for the prosthetic ankle in the transtibial amputee as it is the sole joint that is directly influenced by the biological system (remnant shank) and the mechanical system (prosthetic foot) [4]. Improved cooperation between the person and the prosthesis would then likely decrease stride-to-stride fluctuations to be more similar to the sound leg, possibly resulting in improved patient satisfaction [4]. It would be tolerable to speculate that the prosthesis then that results in improved control is permitting increased coordination of all dynamical degrees of freedom [15], [23].

Examining the effect of appropriateness of the prosthesis, we note the “more appropriate” prosthesis setup did allow for a decreased λ, or reduced stride-to-stride fluctuations at the ankle. This is consistent with the notion that the most appropriate prosthesis is likely to yield dynamics preferred by the patient [4]. Furthermore, this finding agrees with the idea above that the most appropriate prosthesis will allow a patient to achieve stride-to-stride fluctuations that are most similar to the sound leg. In light of this finding, it is difficult not to conclude that effective lower limb loss rehabilitation will reduce stride-to-stride fluctuations as the individual is able to have high coordination of dynamic degrees of freedom [23]. On the other hand, when the device is less appropriate for the individual, these coordinative strategies are not likely to form and therefore there is a higher number of dynamic degrees of freedom needing to be controlled, required increased control and likely an increased risk of negative outcomes. From a dynamical systems perspective, it may be fitting to think of receiving a prosthesis as similar to receiving an organ transplant; a larger system must integrate a vital component into its normal dynamics. Bogaert et al. [24] found when looking at cardiac dynamics no difference between heart transplant recipients and healthy controls. But, Izrailtyan et al. [25] found a shift in the cardiac dynamics amongst those heart transplant recipients that were in the early stages of rejecting the transplanted organ. These two studies seem to highlight that a body can integrate a foreign device/organ into its natural behavior and have near similar dynamics, but when the systems are not cooperating this will reflect in the measured dynamics (e.g. heart beat activity or stride-to-stride fluctuations). Thus, if the prosthetist and limb loss rehabilitation team can properly and effectively prescribe a prosthesis, the outcome will be reduced stride-to-stride fluctuations.

Finally, we wrongly expected a decrease in stride-to-stride fluctuations to occur through the period of adaptation. The idea that the variability in the stride-to-stride behavior would decrease as the person's neuromuscular system learned to use the device is more consistent with the viewpoint that variability arises from noise in the system, and as the person improves control the noise is reduced, leading to decreased variability from stride-to-stride. Rather, what we measured was a learning process previously formulated by Bernstein [26] and since further described [27]-[32]. Bernstein described “the process of mastering redundant degrees of freedom” in which to ultimately arrive upon the optimal movement control. This requires initially freezing a multitude of the degrees of freedom available to the system [31], [32] by creating strong, rigid links. This allows for simplification of the learning task. Then as the task is mastered, there is slow release, or freeing, of the degrees of freedom to increase. The result is a larger movement repertoire allowing for a more flexible and adaptable system [31], [32]. Our design was such that we were able to capture the initial period of high variability due to a lack of coordinative structures and poor control at the initial visit. Specifically, our subjects were fitted with a device and after taking only a few steps (∼60) to allow for proper alignment, we immediately measured the stride-to-stride fluctuations during the treadmill task. At this point, there was an initially increased λ, or increased stride-to-stride fluctuations. When the individual returned 1.5 weeks later, we seemed to be within the period where several dynamic degrees of freedom were frozen as the individual was learning. As a result, there was a significant reduction in the stride-to-stride fluctuations at the prosthetic ankle. When the individual would return for the final visit on the prosthesis, after 3 weeks of wearing the device, the learning had progressed to a stage of freeing up degrees of freedom to increase flexibility and adaptability of the locomoting system. This was captured by a significant increase in stride-to-stride fluctuations at the prosthetic ankle compared to the second visit.

Clinically, it is important to note the lack of statistical difference in the λ at the prosthetic ankle at the initial fitting of the device and after a proper adaptation period. This may indicate the potential to measure stride-to-stride fluctuations with the λ at the initial fitting and not necessarily needing to wait 3 weeks to assess the function of the device. This, however, would need further testing to determine whether this is a statistical finding or whether the λ value truly is similar before and after adaptation. Importantly, the lack of statistical difference though between baseline and post-adaptation does not mean that the mechanism driving the variability from stride-to-stride at the initial fitting and post-adaptation are similar. In fact, the points made previously would rather indicate very different mechanisms: initially increased noise and lack of control compared to ultimate mastery of redundant degrees of freedom leading to greater flexibility and adaptability. Nevertheless, if the initial fitting possibly discloses the stride-to-stride fluctuations expected after adaptation, then it may be possible to use the λ as a means for initial evaluation of prosthesis functionality. Furthermore, future studies measuring λ of joint motion in the lower limb amputee may not need to necessarily incorporate adaptation periods, which can be costly to the study both in terms of monetary funds, time, and potential subject dropout.

There are limitations to this study. First our design setup heavily relied on the subject's prosthetist/physician to have properly classified the patient with regards to their activity level (i.e. K2, K3, or K4). This in itself is problematic for a multitude of reasons, including the ambiguity under which patients are classified [17] and the undeniable fact that the activity level for individuals ambulating with a prosthesis is not possibly four distinct categories but rather represented as a continuum across a spectrum. The only clinical tool available currently to help with patient classification in the Amputee Mobility Predictor [17], but even this tool is known to have large standard deviations making it difficult on the individual level to objectively categorize patients. Furthermore, while we set out to recruit patients from multiple activity levels, specifically K2 and K3 as the break between these levels represents the largest break between prosthesis componentry classifications, we were unable to recruit any individuals that were previously classified as K2 level (“has the ability or potential for ambulation with the ability to traverse low level environmental barriers such as curbs, stairs, or uneven surfaces. Typical of the limited community ambulatory” [17], [33]). While the authors felt there were a few individuals that may have been classified by other providers as K2, our study design was set up such that we would utilize the classification by the subject's prosthetist/physician to improve real world translation. Future work may improve our study design by utilizing a technique to better objectively classify patients, however as it is currently such objective measures do not exist. Furthermore, while we were able to secure multiple high activity feet for the study, with the exception of 1 subject that wore a Walktek foot (K2 foot from Freedom Innovations, Irvine, CA, USA), all low activity feet were SACH feet (The Ohio Willow Wood Company, Mt. Sterling, OH, USA) which helped to improve study logistics (authors were then only needing to acquire high activity feet for each subject once enrolled). As a result, it could be our findings are simply a measured difference between high activity feet and the traditional SACH foot and may not be found in a newer technology K2 (low activity) level foot. However, low activity (or K2) feet are generally more rigid with less flexing and motion, provide a more stable platform for the person to balance on and the functional differences between low activity feet may not be as much as expressed in material costs. The outlined theoretical basis in this manuscript would not seem to support such a simplification of results being limited to the SACH foot. We also see our major findings occurring about the motion of the ankle, which for the majority of prostheses, there is no true ankle joint which could a problem for motion capture [34], [35]. But as noted in Wurdeman et al. [4], it is the deflection and bending about the ankle that recreates flexion/extension, which is the kinematic motion we are measuring.

## Conclusion

The prosthetic leg has increased stride-to-stride fluctuations about the ankle compared to the sound leg, a finding first reported by Wurdeman et al. [2]. In addition, when individuals were fitted with a “more appropriate” and a “less appropriate” prosthesis based on their activity level classification and the prosthesis activity level classification, the “more appropriate” design resulted in decreased stride-to-stride fluctuations. The design that leads to reduced stride-to-stride fluctuations is permitting greater cooperation between the biological system (i.e. amputee) and the mechanical system (i.e. prosthesis) to accomplish the task of walking. When the amputee and the prosthesis are not cooperating and working together, the result is increased stride-to-stride fluctuations as the two systems struggle to operate as a single cohesive unit. Finally, through the course of an adaptation period, the individual's neuromuscular system is undergoing learning as it reconciles the problem of properly integrating a foreign device into its natural movement strategy. Initially this period is characterized by a freezing of the degrees of freedom as the system becomes more rigid [31], [32]. At the end of adaptation, there is a freeing of the degrees of freedom as the system increases its flexibility and adaptability [31], [32].
